# A review of multilayer and composite films and coatings for active biodegradable packaging

**DOI:** 10.1038/s41538-022-00132-8

**Published:** 2022-03-11

**Authors:** Qiankun Wang, Wenzhang Chen, Wenxin Zhu, David Julian McClements, Xuebo Liu, Fuguo Liu

**Affiliations:** 1 College of Food Science and Engineering, Northwest A&F University, 712100 Yangling, Shaanxi PR China; 2 Department of Food Science, University of Massachusetts Amherst, Amherst, MA 01003 USA

**Keywords:** Biomaterials, Biochemistry, Biological techniques

## Abstract

Active biodegradable packaging are being developed from biodegradable biopolymers which may solve the environmental problems caused by petroleum-based materials (plastics), as well as improving the shelf life, quality, nutritional profile, and safety of packaged food. The functional performance of active ingredients in biodegradable packaging can be extended by controlling their release profiles. This can be achieved by incorporating active ingredients in sandwich-structured packaging including multilayer and composite packaging. In multilayer materials, the release profile can be controlled by altering the type, structure, and thickness of the different layers. In composite materials, the release profile can be manipulated by altering the interactions of active ingredients with the surrounding biopolymer matrix. This article reviews the preparation, properties, and applications of multilayer and composite packaging for controlling the release of active ingredients. Besides, the basic theory of controlled release is also elaborated, including diffusion, swelling, and biodegradation. Mathematical models are presented to describe and predict the controlled release of active ingredients from thin films, which may help researchers design packaging materials with improved functional performance.

## Introduction

In recent years, increasing attention has been paid to the problem of environmental pollution caused by the fabrication and disposal of synthetic plastic-based packaging^1^. The food industry is one of the major users of these materials as they are particularly effective at protecting foods from spoilage during storage and transport^2^. For this reason, researchers have focused on the development of innovative biodegradable packaging for application in the food industry to replace plastic packaging^3^. These biodegradable materials are designed to reduce the negative environmental impacts of plastics, while still increasing the shelf life, quality, and safety of packaged food. Biodegradable packaging are typically constructed from food biopolymers (such as proteins and polysaccharides), as well as other functional ingredients (such as lipids, phospholipids, and inorganic particles)^4^. This new generation of packaging is typically greener, safer and more biodegradable than traditional plastics. Moreover, active ingredients such as antimicrobials, antioxidants, and nutrients can be incorporated into biodegradable packaging to gain additional functions (Table 1), such as inhibiting microbial growth, reducing lipid oxidation, and enhancing nutritional value^5^.

**Table 1:** Active ingredients commonly used in food packaging.

Active ingredients		Functions	Stability	Application
Essential oils and plant extracts	Oregano oil	Able to achieve antibacterial, anti-oxidation, antifungal, sweating, and pain relief^101^; improving the barrier properties and mechanical properties of film^102^	Volatile, easy to lose in storage	Pastry^103^; ground beef^104^; beef^105^
	Cinnamon essential oil	Be antimicrobial antifungal, antioxidant, anti-inflammatory, and antidiabetic^106^	Be low solubility, irritations, and allergic reactions^107^	Pork meat balls^108^; dry tofu^109^; strawberry^110^
	Vanilla	Antioxidant and antimicrobial activities, act as a flavor enhancer, cross-linking agent; improving the barrier performance of packaging^111^	thermal instability and volatile nature^112^	Doodhpeda (milk-based solid soft sweet), biscuit, and skimmed milk powder^111^; crab stick^113^; smoked chicken breast^114^
	Green tea extract (Tea polyphenols)	Antioxidant, antibacterial, anti-inflammatory, anti-tumor, and anticancer^115^	Unstable in alkaline and high temperature environments^116^	Pork^117^; marinated anchovies^118^ mushrooms^119^; raw chicken meat^120^
	Tannic acid	Be used as a cross-linking agent, nonsurfactant template, metal chelating ligands; antidiarrheal, astringent and hemostasis, anti-mutagenesis, antivirus and anticancer^121^	Susceptible to oxidation in alkaline solution^122^	Linseed oil^123^; fresh-cut apples^124^
	Carvacrol	Be as flavor and fragrance agent, antioxidant, antibacterial, antifungal, acaricidal, and anticancer^125^	High volatility, low water solubility, and stability^126^	Beef^127^; blackberries and raspberries^128^; ham^129^
Organic acid and their salts	Potassium sorbate	With antibacterial, inhibit mold and corrupt bacteria, hygroscopic	Unstable in air and easily oxidized and colored	Butter cake^130^; soft cheese^131^; lasagna pasta^132^
	Benzoic acid	Be as preservative and flavoring agent^133^	High stability	Cheese and toasted bread^134^;
Enzymes	Lysozyme	Antibacterial; acts as a natural antibiotic, and enhances the efficacy of other antibiotics, strengthens the immune system^135^	Easy to be destroyed by alkali, acid environment; heat stability is very strong	Pork^136^; pear juice and rice milk-based smoothie^137^; ground beef patties^138^
Bacteriocins	Nisin	A wide antibacterial, antifungal, and antiviral activity	Sensitivity to the environmental, stresses, susceptibility to proteolysis^139^; easy to interact with food components such as proteins and fat particles^140^	Beef^141^; ham^142^; hot dog^143^; pork^144^
	Pediocin	Display antimicrobial activity against a wide spectrum of Gram-positive bacteria	Stable in dilute aqueous solutions^145^	Sliced ham^146^; raw sliced pork^147^
Inorganic nanoparticles or microparticles	Silver nanoparticles	Antimicrobial properties against a wide range of microorganisms, including bacteria, yeast, and mould; low effects on the sensory attributes in food^148^	Easy to get aggregation and reaction due to the high surface energy, surface passivating reagent, and capping reagent^149^	Litchi^150^; walnuts, hazelnuts, almonds and pistachios^151^; red grapes^152^
	Titanium dioxide (TiO_2_) nanoparticles	Can be used in food additives, pigments, photocatalysis, and personal care products; for sterilization and industrial photolytic processes regarding the decomposing of organic matters^153^	High chemical stability, biocompatibility, and a robust photocatalytic activity^153^	Cherry tomato^154^; banana^155^; pork^156^; margarine^157^

Nevertheless, biodegradable packaging should be carefully designed to retain these active ingredients and control their release. Many bioactive ingredients may be rapidly released from the film matrix due to their small molecular dimensions, which reduces their efficacy and shortens the shelf life of the food^6^. Thus, it is extremely important to slow down the migration and realize the controlled release of active ingredients during food storage and transportation. The retention and release kinetics depend on the composition and structure of the film matrix^6,7^, while some researchers have achieved controlled release by utilizing multilayer or composite systems^8–11^. However, many studies only focus on the characterization of the macroscopic properties of these materials but ignore the molecular and physicochemical mechanisms that influence their release behaviors. Understanding the underlying mechanisms of controlled release is helpful for researchers in designing new materials and processing methods, which is therefore of great importance to the advance of the food packaging area. For this reason, this paper provides an overview of controllable release from switch structured multilayer and composite active packaging materials, with a focus on the impact of the sandwich structure on controlling the release of active components. The preparation and application of these advanced packaging materials are also reviewed. In addition, the latest advances in this area are summarized, which helps to identify knowledge gaps and to stimulate further research in this important area.

## Advanced packaging with sandwich structure

### Multilayer packaging

**Fig. 1:**
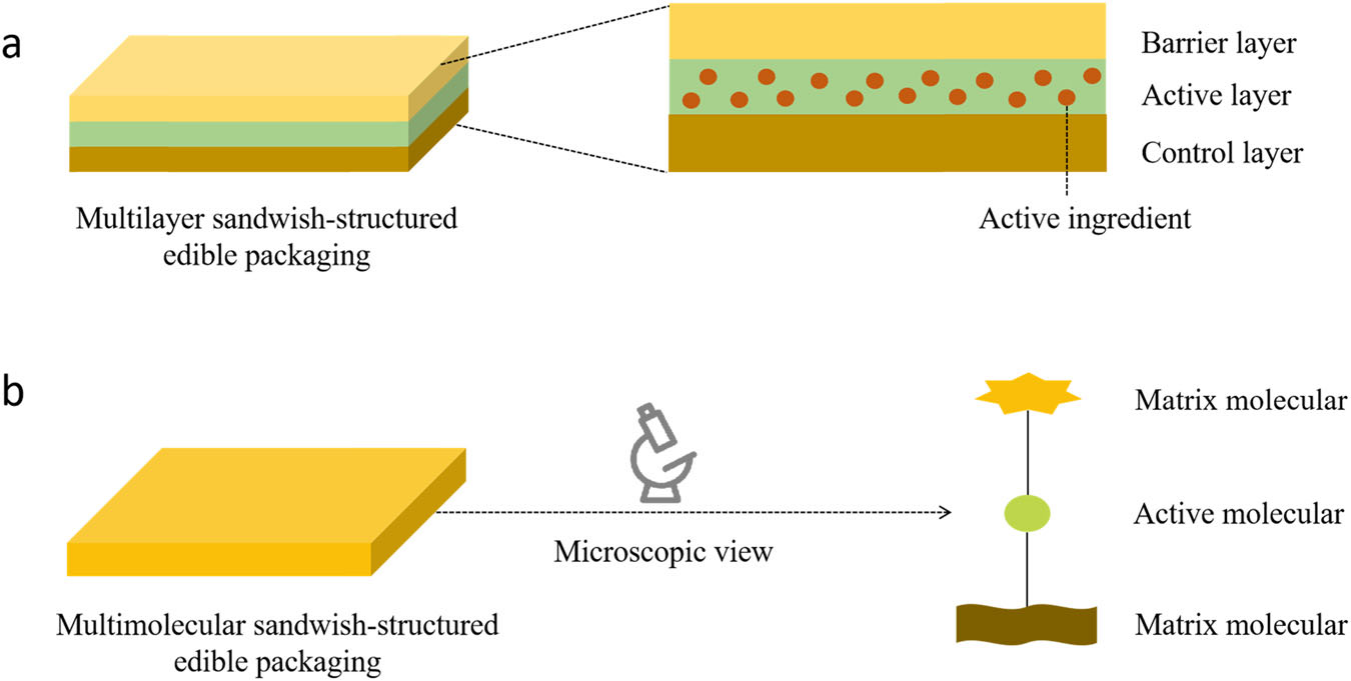
Schematic representation of biodegradable packaging materials with sandwich structure. **a** Multilayer and **b** composite packaging.

The utilization of multilayers often leads to improved or extended functional performance over monolayer materials^12^. This type of packaging material often consists of three distinct layers, which are the barrier, active, and control layers^6^, as shown in Fig. 1a.

The barrier layer is the outermost one in the sandwich-like structure and is therefore in direct contact with the external environment. It is often designed to act as a barrier to substances that cause degradation of packaged food, such as moisture, oxygen, and microorganisms. The barrier layer may also be designed to ensure good retention and protection of the active ingredients located within the interior of the packaging material.

The active layer is located in the middle of the sandwich-like structure and usually contains the active ingredients, such as antioxidants and antimicrobials^13^. It is important to design the system so that the active ingredients are retained in a stable form within the active layer until required, and then diffuse through the control layer into the food at the required rate^7^.

The control layer is the innermost one in the sandwich-like structure, which is directly in contact with food. It is designed to control the rate at which the active ingredients move into the food, as well as to protect the food and active ingredients.

The functional performance of each layer can be controlled by adjusting material properties, such as composition, pore size, thickness, polarity, swelling capacity, and solubility. The release of active ingredients from multilayer packaging can be influenced by various internal and external factors. The internal factors refer to the properties of the biodegradable packaging material itself, including the nature of the active ingredients (such as molecular weight, size, polarity, and concentration) and the properties of the surrounding matrix (such as polarity, thickness, pore size, glass transition temperature, additives, plasticizing, cross-linking degree, crystallization, and swelling)^14–17^. The external factors refer to the influence of the environment on the release profile, including storage time, temperature, relative humidity, and food properties.

Many studies have shown that the structure of multilayer packaging can be manipulated to control the release of active ingredients, so as to prolong their action time. For instance, multilayer biodegradable films have been produced by layer-by-layer solvent-casting using zein as the barrier layer, tea polyphenols as the active layer, and gelatin as the control layer^11^. By changing the ratio of zein/gelatin in the active layer, the water barrier properties and release time of the tea polyphenols could be controlled. The release time of the tea polyphenols from multilayer systems is longer than that from control groups. Other researchers have used electrospinning and coating technologies to prepare multilayer films from polylactic acid (PLA)^18^. These films consist of an active layer containing gallic acid sandwiched between two PLA layers and could be designed to control the release profile of gallic acid into food. Sequential electrospinning has been used to fabricate multilayer films consisting of a curcumin-loaded active layer sandwiched between layers formed from ethylcellulose nanofibers and gelatin nanofibers^19^. The curcumin could be continuously released from these multilayer films and maintain its antioxidant activity throughout this time. Representative examples of research on the controlled release films with sandwich-like structures are summarized in Table 2.

**Table 2:** Representative examples of previous research on degradable multilayer packaging materials with different compositions.

Composition & structure: barrier/active/control layers	Preparation methods	Functional properties
Zein/zein-gelatin-tea polyphenol/gelatin	Layer-by-layer solvent-casting	Tea polyphenol release was slower from multilayer than monolayer films^11^.
Polylactide/gallic acid/polylactide	Electrospinning	Multilayer films could prolong polyphenol release for more than 1000 h^18^.
Ethylcellulose/gelatin-curcumin/ethylcellulose	Sequential electrospinning	Multilayer films released curcumin continuously for 96 h and maintained its antioxidant activity^19^.
Cellulose acetate/potassium sorbate/cellulose acetate	Dry phase inversion technique	Potassium sorbate release was slower from multilayer than monolayer films^28^.
PHBV/zein-cinnamaldehyde/PHBV Alginate/zein-cinnamaldehyde/PHBV	Electrospinning	Multilayer films could be designed with good antibacterial activity^67,158^.
Chitosan/chitosan-cinnamon oil/sodium alginate	Layer-by-layer electrostatic deposition technique	Multilayer coatings had better antimicrobial activity than monolayer coatings^68^.
Sodium alginate/chitosan-cinnamon essential oil/sodium alginate	Layer-by-layer solvent- casting	Multilayer films had better retention and sustained release than monolayer films^68^.
Balangu seed gum/gelatin-menthol/balangu seed gum	Electrospinning	Multilayer films were designed to control the release of menthol^159^.
PUR/PVA-gentamicin/PUR	Needleless electrospinning technology	Multilayer films with good antimicrobial activity could be designed^66^.
Zein/zein-thymol/zein Zein-spelt bran/zein-hymol/zein-spelt bran	Layer-by-layer solvent-casting	The thymol release rate could be controlled by altering film thickness and bran content^160^.
Zein-gelatin/zein-gelatin-oregano oil/zein-gelatin	Continuous casting method	Tri-layer films with oregano oil in intermediate and/or upper layer exhibited a high retention rate^161^.
Alginate/chitosan-sodium benzoate alginate beads/alginate	Layer-by-layer solvent-casting	Alginate beads could be used to control the release rate of sodium benzoate^162^.
PVOH/PVOH-lysozyme/PVOH	Layer-by-layer solvent-casting	The release rate of lysozyme from the films could be controlled^163^.
Bacterial cellulose/PVA-bacterial cellulose-sorbic acid/bacterial cellulose	Layer-by-layer assembly	Sorbic acid release was slower from multilayer than monolayer films^164^.
Bacterial cellulose/PVA-bacterial cellulose-vanillin/bacterial cellulose	Layer-by-layer solvent-casting	A bacterial cellulose control layer delayed vanillin release and prevented PVA dissolution in food^165^.
Starch/PCL-carvacrol/starch	Electrospinning	Multilayer films prolonged antimicrobial action and reduced water vapor permeability compared to starch films^86^.
Bacterial cellulose/ bacterial cellulose-*Scrophularia striata*/β-cyclodextrin	Layer-by-layer solvent-casting	The release rate of *Scrophularia striata* was reduced in food simulant with multilayer films^166^.

### Composite packaging

This type of packaging material is comprised of a blend of active and matrix molecules that are linked together by covalent or noncovalent bonds, such as electrostatic, hydrophobic, hydrogen bonding, or van der Waals interactions^20^, as shown in Fig. 1b. Covalent bonds involve the sharing of electron pairs between two atoms^21^. As a result, they tend to be stronger and more stable than noncovalent bonds^22^. The formation of new covalent bonds between the molecules used to form packaging is usually achieved through specific chemical or enzymatic reactions^23^. Some of the most commonly utilized reactions, such as esterification, etherification, amidation, and glycosylation, occur between particular functional groups on active and matrix molecules^24^. Noncovalent bonds also rely on the presence of particular functional groups on these molecules, such as the nonpolar groups for hydrophobic interactions, polar groups for hydrogen bonding interactions, and charged groups for electrostatic interactions^20^.

The bonds formed between the active and matrix molecules restrict the migration of the active ingredients in the packaging, which can be used to control their retention and release^25^. As an example, the formation of bonds between thyme extract polyphenols and the chitosan/pea starch in composite films has been reported to reduce the release rate of polyphenols^26^. Furthermore, the incorporation of tannic acid into the films could reduce polyphenol release by cross-linking the chitosan molecules in the biopolymer matrix. The release rate of active molecules from this kind of packaging material can also be modulated by altering the properties of the film matrix, such as the porosity, swelling, and biodegradation^6^. In another study, composite packaging was assembled from two active ingredients (tea polyphenol and oregano oil) and two matrix molecules (zein and gelatin), which was designed to control the release of active ingredients^27^. It was reported that hydrogen and hydrophobic bonds were formed between these active ingredients and matrix molecules. The microstructure of the films changed after the active ingredients were incorporated, with evidence of small spherical droplets being dispersed within a biopolymer matrix. Other examples of research on the retention and release of active ingredients from composite packaging are summarized in Table 3.

**Table 3:** Advanced examples of active degradable packaging materials assembled from sandwich-like molecules.

Matrix composition	Active ingredients	Perspectives
Chitosan, pea starch	Thyme extract polyphenols	The interaction between polyphenols and the matrix inhibited polyphenol release; solvent polarity affected polyphenol release and antioxidant activity of the film^26^.
Zein, gelatin	Tea polyphenol, oregano essential oil	Hydrogen and hydrophobic bonding occurred between active ingredient and the matrix; the retention of active ingredient promoted by simultaneous loading of tea polyphenols and oregano oil in composite film^27^.
Pullulan, gelatin	Potassium sorbate	The release mechanism of potassium sorbate depended on dissolution and swelling of matrix; release rate could be adjusted by changing pullulan and gelatin ratio^167^.
Oligomeric proanthocyanidins, gelatin	Lysozyme	Increasing oligomeric proanthocyanidin cross-linking retarded lysozyme release^78^.
Cinnamaldehyde, gliadin	Lysozyme	Increasing biopolymer matrix cross-linking retarded lysozyme release^79^.
Methylcellulose, glutaraldehyde	Maqui (*Aristotelia chilensis*) berry fruit extract	Maqui extracts reacted with glutaraldehyde through phenolic or glycoside-hydroxyl groups; the release amount of antioxidant compounds increased with increasing glutaraldehyde concentration^80^.
Soy protein isolate/poly(ethylene oxide) blend, poly(lactic acid)	Allyl isothiocyanate	The release of allyl isothiocyanate could be controlled by changing the relative humidity^168^.
Low methoxyl pectin	Lysozyme	Low methoxyl pectin formed insoluble complexes with lysozyme, mainly due to electrostatic attraction, which controlled the release of antimicrobial lysozyme^169^.

## Preparation of advanced packaging

### Preparation of multilayer packaging

The preparation of packaging materials with the appropriate functional attributes requires the selection of appropriate ingredients, film-forming techniques, and preparation conditions^12,28^. This section describes the main preparation technologies and conditions that are typically used to prepare biodegradable packaging materials with different structures and functional properties. Based on literature, the most widely used approaches for preparing multilayer packaging materials are the layer-by-layer (LbL), electrohydrodynamic, and coextrusion methods, while the most widely used ones for creating composite packaging materials are extrusion and solution casting.

#### Layer-by-layer (LbL) assembly

**Fig. 2:**
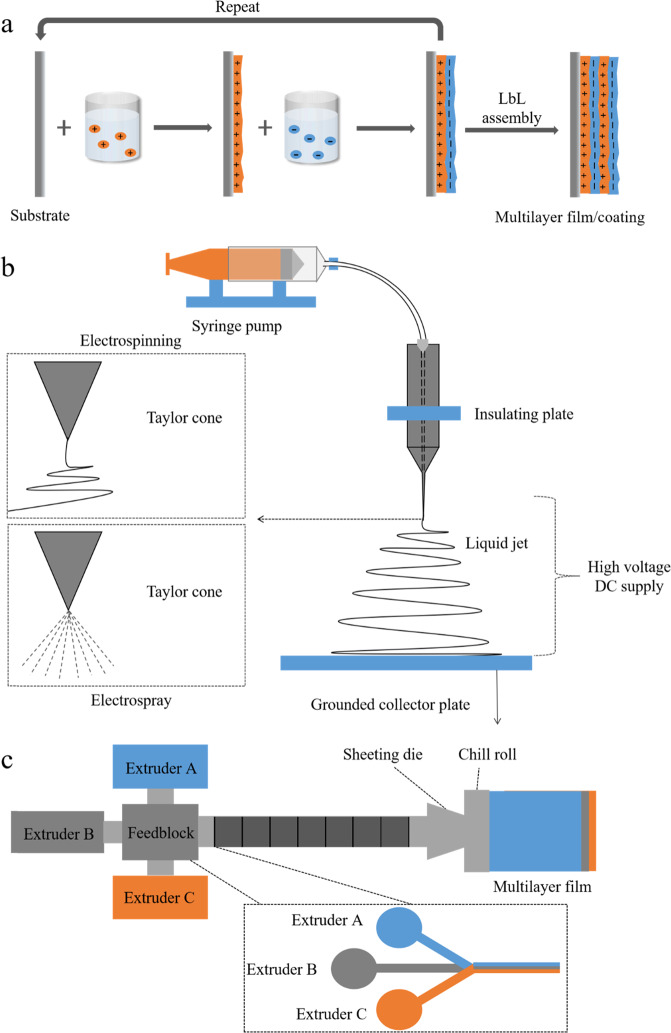
Preparation of multilayer edible films. **a** Electrostatic layer-by-layer (LbL) deposition. **b** Electrospinning or electrospraying. **c** Coextrusion.

This is a method based on electrostatic attraction, hydrogen bonding, hydrophobic attraction, or entanglements between molecules in neighboring layers. In general, this method involves the formation of multilayer films or coatings by sequential deposition of numerous film-forming materials onto a surface^29^. The utilization of electrostatic attraction between successive layers is one of the most commonly used methods for assembling this type of film or coating (Fig. 2a). It involves immersing a substrate with a negative surface charge into a solution containing positively charged substances, which causes these substances to be attracted to the surface of the substrate. After washing off the excess solution, the positively charged substrate formed is then immersed in another solution containing negatively charged substances, which causes these substances to form a new layer. This process can be repeated a number of times to produce multilayered films or coatings^30^. Typically, the materials used in this electrostatic LbL deposition process are water-dispersible and electrically charged biopolymers or colloidal particles. The properties of the films or coatings formed (such as their composition, thickness, and charge) can be manipulated by altering the type, number, and sequence of the charged substances used to assemble the layers.

In addition to electrostatic attraction, other intermolecular interactions can also be used to assemble LbL films or coatings, such as hydrogen bonding, covalent bonding, and hydrophobic interactions^9^. The interactions involved depend on the nature of the substances used to prepare the biodegradable packaging. For instance, proteins often form cross-links with neighboring molecules through a combination of hydrophobic, electrostatic, covalent, and hydrogen bonds^31,32^.

The LbL method has a number of potential advantages over other fabrication techniques for preparing multilayer packaging^29^:

1. The preparation process is simple and inexpensive, requiring no specialized equipment;
2. Films with different functional attributes can be created by altering their physicochemical and structural properties (such as composition, thickness, and charge) by selecting different types, numbers, and sequences of film-forming materials;
3. The preparation of multilayer films or coatings is not limited to flat surfaces and can be applied to food with different shapes.

#### Electrohydrodynamic process

The electrohydrodynamic process involves the spraying of polymer solutions into microscale or nanoscale fibers or particles by applying a high-voltage electric field. There are two common methods used in the electrohydrodynamic process: electrospinning and electrospraying^33^. The packaging formed using electrospinning tends to have a high porosity, high polymer orientation, and large specific surface area, which is advantageous for some food applications^34^. The utilization of electrospinning to form the layers in multilayer packaging has been shown to enhance the mechanical, optical, and/or functional properties^35^.

The apparatus typically used to create packaging using the electrospinning technique consists of four main parts: a high-voltage power supply, an injection pump, a capillary tube with a tip, and a metal collector (Fig. 2b). In detail, a polymer solution is placed into the capillary tube and then a high-voltage electric field is applied between the tip of the tube and collection plate. The polymer solution will be drawn out of the tip and form a jet of fluid in the form of a twisted Taylor cone. During its passage from the tip to the collection plate, the solvent is rapidly evaporated, which leads to the formation of polymer-rich nanofibers that are deposited onto the collector^34,36,37^.

Electrospraying works in much the same way as electrospinning, but the polymer solution and operating conditions used are designed so that the Taylor cone jet produced remains stable and does not lengthen^38,39^. As a result, nanoparticles are formed by electrospraying rather than the nanofibers formed by electrospinning. It has been reported that electrospraying is more suitable for preparing biodegradable coatings, while electrospinning is more suitable for preparing biodegradable films^33^. Compared to other methods of preparing packaging, electrohydrodynamic methods have the following potential advantages:

1. Polymer nanofibers can be prepared directly, continuously, and on a large scale, which is beneficial for commercial applications;
2. The conditions used to form films or coatings are relatively mild, allowing a broad range of raw materials utilized;
3. The production equipment is relatively inexpensive and easy to operate (but caution must be taken because of the high voltages used);
4. The morphology and functional performance of the films or coatings produced can be manipulated by altering the ingredients and processing operations used;
5. Fibers or particles with nanometer-scale dimensions can be fabricated, which leads to films or coatings with large specific surface areas and high porosities.

#### Coextrusion

Films with multilayer structures can be prepared using the coextrusion method by heating two or more different kinds of polymer materials above their glass transition temperature and then extruding them separate through a specially designed nozzle^40^. The coextrusion process generally includes four main steps: feeding, melting, stream confluence, and coextrusion. Based on the way that the different polymer streams are brought together in the film-forming process, coextrusion can be divided into two main types: die and feed block^12^. In die coextrusion, two or more materials are fed separately into the extruder and heated, and then the different material melt streams are brought together at the exit of the die (Fig. 2c). In feed block coextrusion, two or more materials are brought together before the extrusion die, then made to form a laminated layer of melt stream, which is then extruded through the die. Coextrusion has been widely used for the industrial production of polymer-based packaging. Compared to other technologies, coextrusion has the following potential advantages:

1. It is beneficial for commercial applications with relatively short processing time and low energy consumption;
2. Powdered film-forming materials can be used as the input to the extruder, which means that there is no need to remove any solvents after film formation;
3. The range of operating conditions is relatively wide, which means that film-forming materials with different melting points can be utilized;
4. The mechanical and optical properties of biodegradable packaging can be improved^41,42^;
5. The mixing degree of material and the thickness of films formed can be accurately controlled^43^.

### Preparation of composite packaging

**Fig. 3:**
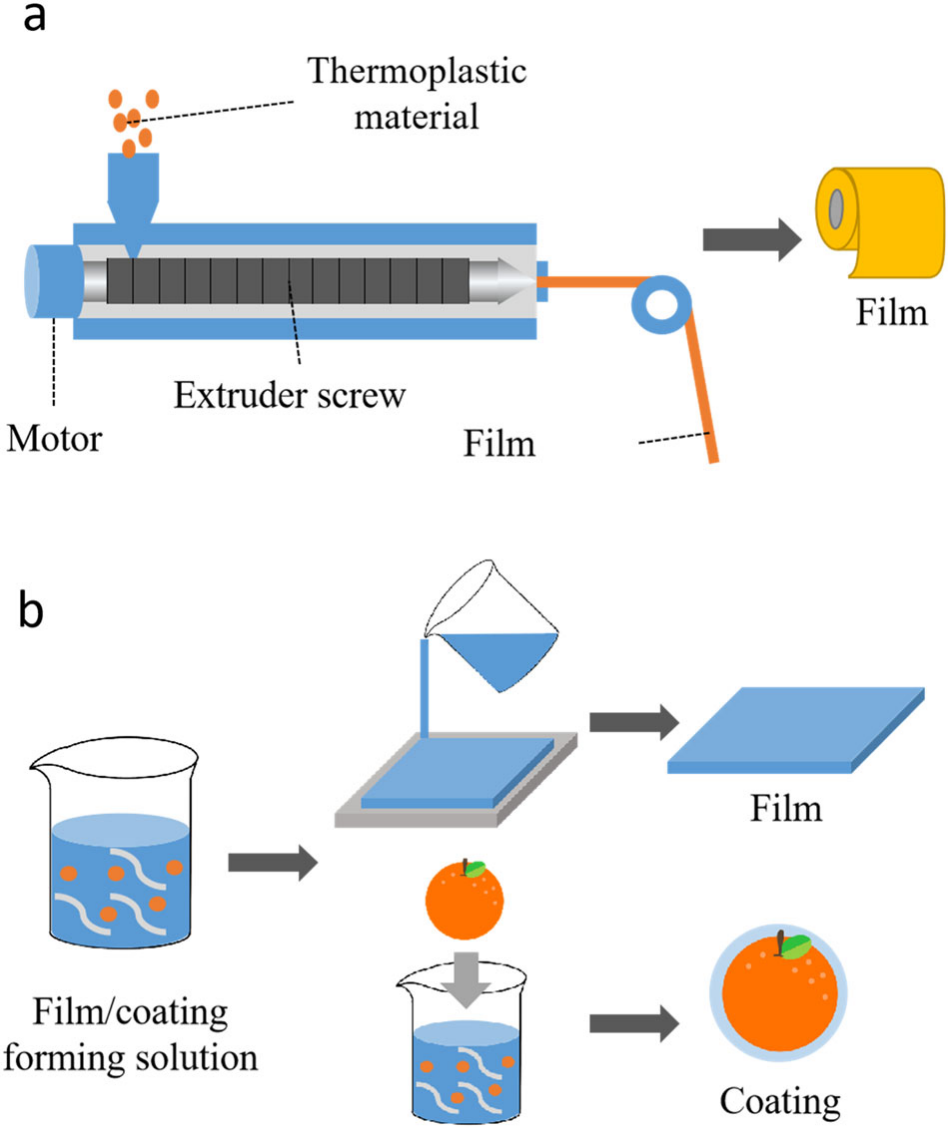
Preparation of composite edible films/coatings. **a** Extrusion. **b** Solvent-casting.

This type of biodegradable packaging material typically consists of active and matrix molecules^44^. In this case, the whole of the packaging material can therefore be regarded as a carrier system for the active ingredients. The structure and properties of these films can be controlled by altering the composition and processing methods used to fabricate them, which leads to different functional attributes. In general, there are two major methods that are currently used for preparing composite biodegradable films: the dry and wet methods^45^. Typically, extrusion is used to produce films from solids, whereas solvent-casting is used to produce films from solutions (Fig. 3)^5^.

#### Extrusion

The extrusion method mainly utilizes the thermoplastic properties of polymer materials to prepare packaging. In this method, a solid material with low water content is heated above its glass transition temperature to form a melt, thoroughly mixed, and then passed through a suitably-shaped nozzle. The resulting material is then cooled leading to the formation of a film. The use of the extrusion method to prepare biodegradable films typically involves several steps: feeding, mixing, hydration, and extrusion. Thermoplastic food materials, such as powdered polysaccharides (e.g., starch or wheat gluten)^46,47^ and proteins (e.g., zein, soy protein, whey protein, or gelatin) are typically used as starting materials for extrusion^41,42,48^.

The various steps in the extrusion process are briefly summarized here^42^. First, the polymers and plasticizers are introduced into the feed zone and air compression is used to reduce the moisture content of these materials. The materials then enter the kneading zone where they are subjected to a high-temperature/high-pressure kneading treatment. They are then heated to above the glass transition temperature to convert into a melt form, which is extruded through the nozzle due to forces generated by the rotation of the extrusion screw. Unlike coextrusion, the polymer materials used to produce films that are mixed and not layered during the fabrication process. Compared to the other film-forming methods, extrusion has a number of potential advantages, including high throughput, low cost, continuous production, and the ability to process a wide range of materials.

#### Solvent-casting

Solvent-casting is the most commonly used method for preparing biodegradable films in laboratories. It generally includes three steps: solution preparation, mold casting, and drying^49^. Since it is difficult to form a biodegradable film with all the functional properties required using a single material, solution preparation usually involves selecting and mixing multiple film-forming materials and functional additives together in an appropriate solvent. The various components used may be molecules dissolved in solution or particles suspended in solution. This process is typically carried out at room temperature but may be carried out at other temperatures if required. The resulting system is then poured into a suitable mold and the solution is dried by removing the solvent through evaporation, which may involve simple air-drying or the use of drying equipment. During the drying process, the interactions and arrangement of the molecules in the system will change. Compared to the extrusion method, the solvent-casting method does not require the use of complicated equipment or expensive cost, and the preparation process is simple. At the same time, the absence of high-temperature heating avoids the degradation of heat-sensitive active ingredients. However, the preparation process of the solvent-casting method takes a long time and is not suitable for mass production^50^.

## Controlled release types and mathematical models for active packaging

The release of active ingredients from food packaging may involve controlled or sustained release mechanisms, which can be achieved by regulating the parameters affecting the movement of the active molecules through the film matrix. In general, the release rate is influenced by the nature of the active ingredient, the properties of the matrix materials, the film-forming method used, the properties of the release media, and the environmental conditions. The main objective of sustained release is to prolong the release of the actives from the film so that the desired activity is maintained over an extended period. The main objective of controlled release is to create a specific release profile that depends on the application, which could involve, burst, triggered, or sustained release. Researchers in this area aim to establish the impact of specific active and matrix properties on the release profile so that they can manipulate it for the required application^51^.

### Diffusion-controlled release

**Fig. 4:**
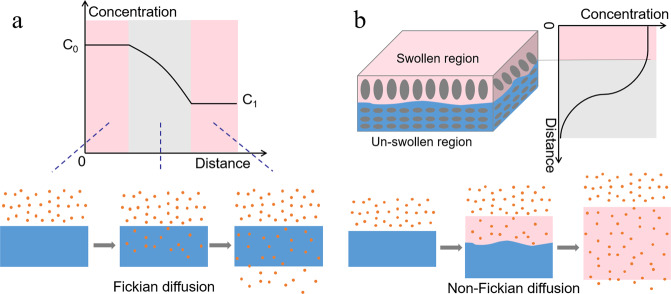
Distribution models of active ingredients in the film/coating during diffusion. **a** Fickian model **b** non-Fickian model.

Diffusion refers to the process where active ingredients in food packaging move from areas of high to low concentration as a result of the concentration gradient. Diffusion is present in all controlled release systems, and only systems with diffusion as the dominant release mechanism are referred to as “diffusion-controlled” release systems. Diffusion-controlled release is usually described by Fick’s law (Fig. 4). When the relaxation time of the matrix material is much longer than the characteristic solvent diffusion time, the controlled release system conforms to Fick’s law^51^. It should be noted that Fick’s law only applies to simple shaped systems where time, space, and concentration are independent of diffusivity. Unlike the erosion and biodegradation mechanisms, the degradation of matrix materials is not obvious during diffusion release. Typically, this diffusion release mechanism can be implemented using either reservoir or matrix systems.

**Fig. 5:**
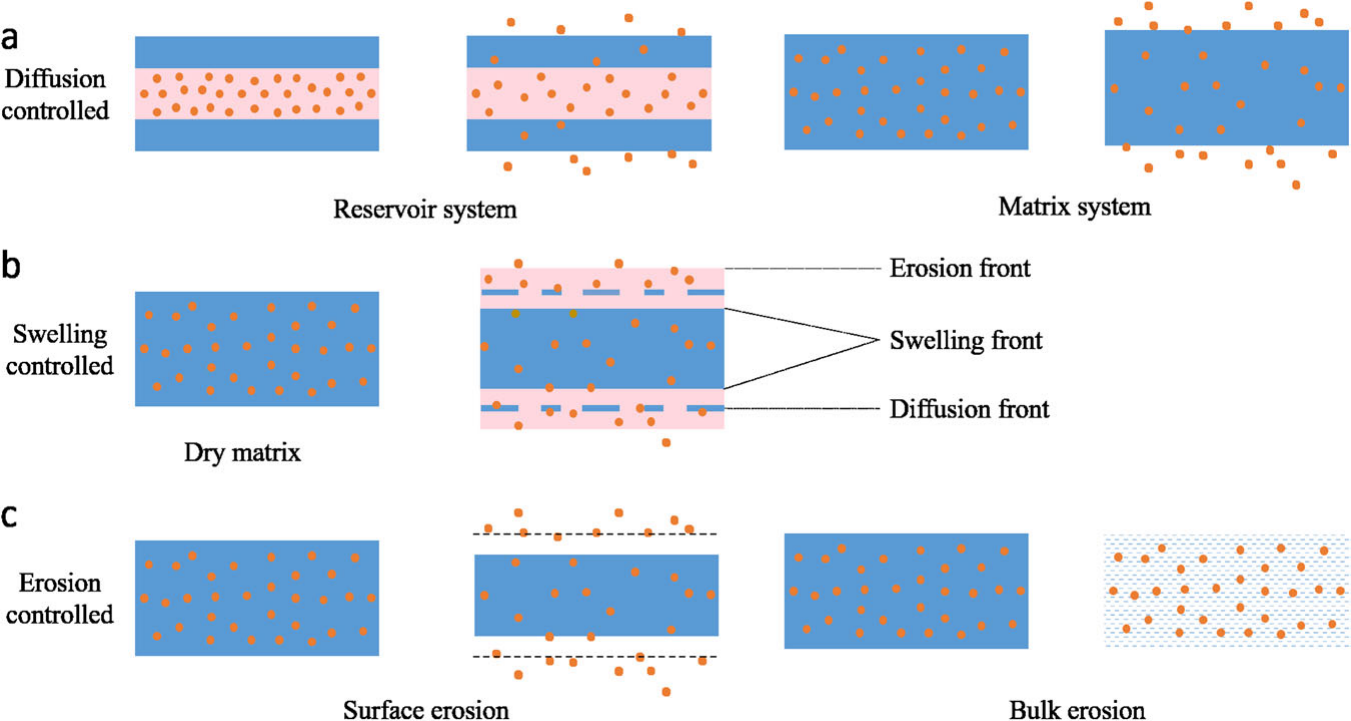
Three common release mechanisms utilized in active packaging. **a** Diffusion-controlled release. **b** Swelling-controlled release. **c** Erosion-controlled release.

The reservoir system typically consists of an active layer and a barrier layer, with the active ingredient located inside the active layer, as shown in Fig. 5. In a reservoir system, the diffusion rate of the active ingredient depends on the properties of the barrier layer, including its thickness, area, and permeability^7^. Generally, the release of active ingredients involves three processes: the diffusion of solvent, the dissolution of active ingredients and the diffusion of active ingredients. In the case of ignoring the boundary layer resistance, the release rate is mainly controlled by the active ingredient diffusion step. When there is enough active ingredient in the packaging material, diffusion follows zero-order kinetics, that is, the active ingredient maintains a constant release rate, thus achieving the purpose of controlled release^52^.

Under appropriate conditions, Fick’s first law of diffusion can be used to describe the release of active ingredients from reservoir systems using the following mathematical model^19^.

1 $$\frac{{{\mathrm{d}}M}}{{{\mathrm{d}}t}} = \frac{{ADK{\Delta}c}}{L}$$

Here, d*M*/d*t* is the release rate of the active ingredient at time *t*, and *A* and *L* are the surface area and thickness of the layers respectively. *D* is the diffusion coefficient through the matrix material; *K* is the distribution coefficient of the active ingredient between the film and environment; and Δ*C* is the solubility of the active ingredient. When *D, L, K*, and Δ*C* remain unchanged, the Δ*M*/d*t* is stable, which results in a constant release rate of the active ingredients. When the active ingredient is released, it may affect the integrity of the barrier layer, causing collapse or cracks and resulting in changes in the surface area, thickness, or permeability of the material, and the release process no longer conforms to the zero-order release kinetics, making the release rate unstable^53^. Therefore, the selection of barrier materials in the reservoir system is very important for the controlled release of active ingredients. Generally, materials that are not easily degraded or swelled are selected as the barrier layer.

In the matrix system, the active ingredients are uniformly distributed within a non-biodegradable and non-erodible matrix. The release rate is related to the shape of the system, the type of matrix material used, and the properties of the active ingredient. In a matrix system, the release steps of the active ingredient include dissolution of the active ingredient in the matrix, diffusion of the active ingredient through matrix, and the removal of active ingredient from the surface of the matrix. Among them, the rate-limiting step for the release of active ingredient is usually diffusion through the matrix. Since there is no barrier layer, the matrix system will have a burst release in the early stages, and then the release rate will gradually decrease over time. The release rate of active ingredients in the matrix system generally follows Fick diffusion, and the release rate varies with the shape of the system. The shape of the system can be assumed to be an infinite flat plate with a thickness of *δ*, and the change of the release rate of active ingredients with time can be described by the following mathematical model^54^.

2 $$\frac{{M_t}}{{M_\infty }} = 1 - \mathop {\sum}\nolimits_{n = 0}^\infty {\frac{8}{{(2n + 1)^2\pi ^2}}exp\left[ {\frac{{ - D_m(2n + 1)^2\pi ^2t}}{{\delta ^2}}} \right]}$$

Here, *M*_*t*_*/M*_∞_ represents the fractional solute release of the active ingredient at time *t*, and *D*_m_ represents the diffusion coefficient of the active ingredient through the matrix. At the initial stage of the release, when the fractional solute release is less than 60%, the release rate of the active ingredient is given by the following mathematical model:

3 $$\frac{{M_i}}{{M_\infty }} = 4\left( {\frac{{D_{ip}t}}{{\pi \delta ^2}}} \right)^{1/2}$$

In this formula, *M*_*t*_*/M*_∞_ is proportional to *t*^−0.5^, thus it is difficult to maintain a stable release rate of the active ingredient in the matrix system with a simple flat plate structure. Instead, controlled release from matrix systems is often achieved by controlling the shape of the matrix structure, e.g., cylindrical or spherical^55,56^. In contrast, reservoir systems vary the properties of the barrier layers to control the release of active ingredients, e.g., their thickness or material properties. Compared to the matrix system, the reservoir system is easier to obtain stable diffusion rates, so it is more widely used in food active packaging.

### Swelling-induced release

Swelling-controlled release systems are based on changes in the morphology of the polymer matrix when it comes into contact with certain solvents. In this case, when the matrix contacts with the solvent, it absorbs solvent molecules, which causes it to swell. This process increases the spaces between the polymer molecules in the matrix, thereby allowing the active molecules to be released. More specifically, the matrix material undergoes a glass transition when it absorbs solvent molecules, which changes the structure of the matrix and dissolves active ingredients. As a result, the diffusion coefficient of active ingredient increases, which allows it to move through the polymer matrix and be released into the surroundings. During this process, the matrix material is not degraded or eroded, so the packaging material maintains its original shape. The process of swelling-controlled release systems therefore involves several steps: solvent absorption, volume swelling, active dissolution, and active diffusion^19^.

The release rate depends on which step is the rate-limiting one. If the solvent absorption, dissolution or diffusion steps are the rate-limiting steps, the release of active ingredient conforms to Fickian diffusion. Conversely, if the volume swelling step is the rate-limiting one, the release of the active ingredient is non-Fickian diffusion. In this case, the relevant parameters of the volume swelling need to be considered^57^.

After the matrix absorbs the solvent, it undergoes a transition from the glassy to the rubbery state, which increases the molecular mobility of the polymer chains and the porosity of the network structure. This process is usually described by the following simple mathematical model^58^:

4 $$\frac{{M_t}}{{M_\infty }} = kt^n$$

In this formula, *M*_*t*_*/M*_∞_ represents the fractional solute release of the active ingredient; *k* represents the rate constant; and *n* is a number that depends on the release mechanism: 0.5 < *n* < 1 for non-Fickian diffusion; *n* = 0.5 for Fickian diffusion (as shown in Eq. (3)); *n* = 1, Fickian and non-Fickian diffusion occur simultaneously, which is called case II transport^7^. When the release rate of the active ingredient only depends on the swelling rate of polymer, the release rate of the active ingredient is positively correlated with the amount of solvent entering the system.

For non-Fickian diffusion, the following mathematical model of expansion dynamics can be used considering the influence of polymer viscosity on osmotic pressure, assuming the sheet plate model^59,60^:

5 $$\frac{{dV}}{{dt}} = \frac{{ - RT}}{{V_1N_AKG}}\ln \left( {\frac{A}{V}} \right)$$

In this formula, *V*_1_ is the molecular volume of the solvent; N_A_ is the Avogadro constants; *V* is the concentration of the active ingredient; *A* is the activity of the solvent; and d*V*/d*t* is the concentration of the active ingredient. By numerical integration of this equation, the function of the swelling part of the glassy polymer in the thin plate with time can be obtained. On this basis, it has been reported that the release of active ingredients depends on the dynamic change of substrate thickness. For instance, for the swelling of matrix tablets, either erosion or diffusion front motion could inhibit the release kinetics of active ingredients, depending on their relative rates^61^. The matrix materials used in swelling-controlled release systems are generally hydrophilic polymers such as proteins or polysaccharides that can undergo glassy to rubbery transitions in the presence of solvents (typically water).

### Degradation-induced release

Degradation refers to the process of chemical degradation of polymer molecules and/or physically disruption of polymer matrix, which leads to the simultaneous breakdown of packaging and release of active ingredients^62^. When the substrate is a biodegradable material and the erosion rate of the substrate is much less than the diffusion rate, the diffusion is considered as the rate-limiting step, and the release kinetics of the active ingredients is mainly characterized by diffusion, which is described by Fick’s law (see Eqs. (1–3)). Conversely, when substrate erosion is the main rate-limiting step, the controlled release rate of active ingredients is mainly affected by the degradation rate of matrix materials^54^. Depending on the degradation mechanism, either surface or bulk erosion can occur^63^.

Surface erosion involves degradation that occurs only at the surface of polymer matrix that is in direct contact with the surrounding solvent, and often involves polymers that contain functional groups that undergo rapid hydrolysis^63^. The release rate of active ingredients is affected by the shape and surface area/volume ratio of system. In the absence of boundary effects, the release rate of active ingredients from flat films is given by:

6 $$\frac{{{\mathrm{d}}M}}{{{\mathrm{d}}t}} = {{{\mathrm{C}}}}_0{{{\mathrm{BA}}}}$$

In this equation, d*M/*d*t* is the release rate of the active ingredient; C_0_ represents the content of the active ingredient per unit area; B represents the surface degradation rate of the polymer; and A is the surface area of the film. When the surface area remains constant, a steady release rate of active ingredient can be achieved by ensuring that C_0_*B also remains constant. In this case, the degradation rate of polymer surface should be inversely proportional to the content of active ingredient per unit area.

When C_0_*B is constant, the dM/dt is proportional to the surface area A, which is in line with the Hopfenberg empirical model^64^:

7 $$\frac{{M_t}}{{M_\infty }} = 1 - \left( {1 - \frac{{k_0t}}{{C_0a}}} \right)^n$$

In this formula, *M*_*t*_*/M*_∞_ represents the fractional solute release of the active ingredient; *k*_0_ is the erosion rate constant; *C*_0_ is the initial concentration of the active ingredient in the system; *a* represents 1/2 of the slice thickness; and *n* is related to the system’s shape: for a thin slice, *n* = 1; for a cylinder, *n* = 2; and for a sphere, *n* = 3. Since the Flake model is mainly used in food packaging, *n* = 1. Consequently, the release rate can be described by the following expression:

8 $$\frac{{M_t}}{{M_\infty }} = \frac{{k_0t}}{{C_0a}}$$

In this case, the release of active ingredients conforms to zero-order release kinetics, so that a controlled release of active ingredients can be realized.

When the diffusion rate of solvent in the system is greater than the degradation rate of the matrix, then the degradation mode is bulk erosion. In this case, the solvent uniformly disperses throughout the polymer matrix, causing the polymer chains to break or dissociate and the system to erode uniformly^7^. The release rate of the active ingredient is not related to the surface area or volume of the system but depends on the solvent diffusion rate and the decomposition/dissociation rate of the polymer. Generally speaking, the release rate in the early stage of bulk erosion is relatively small, then increases rapidly with the rapid diffusion of the solvent and the continuous degradation of the matrix.

However, both surface and bulk erosion mechanisms are in ideal conditions. In practice, polymer matrices are degraded or dissociated by a mixture of both surface and bulk erosion. In this case, the situation is more complex, and an erosion parameter Ɛ can be defined^63^:

9 $$\varepsilon = \frac{{\langle x\rangle^2\lambda \pi }}{{4D_{eff}\left( {\ln \left[\langle x\rangle\right] - \ln \left[ {\root {3} \of {{\overline {M_n} /N_A\left( {N - 1} \right)\rho }}} \right]} \right)}}$$

In this model, $\langle {{{\mathrm{x}}}}\rangle$ represents the average diffusion distance; λ is a constant; *D*_*eff*_ is the effective diffusivity of water in the polymer; $\overline {M_n}$ is the average molecular weight of the polymer; N_A_ is the Avogadro constant; *N* is the average number of monomers per polymer chain; and ρ represents the density of the polymer matrix. It can be seen from this equation that the type of polymer and the shape of the system are the main factors affecting the release rate of the active ingredients. When applying this model to food packaging, the value of $\langle {{{\mathrm{x}}}}\rangle$ can be manipulated by adjusting the thickness of the film and changing the polymer type to achieve the appropriate erosion parameters.

When the polymer is hydrophilic or contains highly reactive functional groups, water can rapidly spread throughout the polymer matrix, leading to predominantly bulk erosion. Conversely, when the polymer is hydrophobic or contains less reactive functional groups, it is more prone to surface erosion^52^. One of the main advantages of the surface erosion mechanism is that the release rate can be controlled by altering the surface area of matrix material^54^.

## Controlled release mechanisms of advanced active packaging

### Multilayer packaging

The controlled release of active ingredients in food packaging depends on many factors, with the most important ones depending on the dominant release mechanism. The composition and structure of packaging can be controlled to manipulate these factors. In this section, we consider the use of packaging with multilayer structures, which have been shown to be better at controlling the release of active ingredients^65^. The mechanisms of which multilayers can improve the release characteristics of films are described in the following text.

There are more options for controlling the release properties of multilayer films than other films. For instance, the composition, properties, and thickness of each of the different layers used can be manipulated. Initially, we consider the impact of increasing the thickness of the layers. In diffusion-induced release reservoir systems, it can be seen from Eq. (1) that the film thickness (L) is inversely proportional to the release rate. In swelling-induced release systems, the release of active ingredients depends on the increase in the film thickness over time. In the biodegradable-release induced system, the average diffusion distance of active ingredients $\langle {{{\mathrm{x}}}}\rangle$ in Eq. (9) affects the erosion parameters of the system. Thus, the distance between the active ingredients and food surface affects their release profile. The impact of layer thickness on the release profile of gentamicin from multilayer materials (PUR/PVA/PUR) assembled from polyvinyl alcohol (PVA) and polyurethane (PUR) nanofibers have been studied^66^. The results show that the retention time of gentamicin could be prolonged by increasing the thickness of the layers. In another study, the release of potassium sorbate from cellulose acetate/potassium sorbate/cellulose acetate (CA/Psb/CA) multilayer films was compared to that from the monolayer films^28^. The release rate of potassium sorbate is slower from the multilayer films than the monolayer ones. In the monolayer films, potassium sorbate release is controlled by Fickian diffusion at the beginning but then dissolution of potassium sorbate crystals at the later stages. In contrast, for the multilayer films, there are no crystals present and so the release is only controlled by diffusion.

In a sandwich-like multilayer structure, the protective effect of barrier layer can reduce the loss of active ingredients, which can improve the retention and activity of active ingredients. In another study, cinnamaldehyde was loaded into multilayer materials formed from zein, poly(hydroxybutyrate-co-hydroxyvalerate) (PHBV), and/or sodium alginate films^67^. The cinnamaldehyde was trapped in the zein layer. The release rate and release mechanism of the cinnamaldehyde could be manipulated by altering the number and type of additional layers used. In particular, the release rate decreased as the number of layers was increased. In another study, cinnamon oil (CO) was encapsulated within multilayer materials assembled from chitosan (CS) and sodium alginate (SA) films^68^. The release profile of this essential oil was compared for monolayer films (CS–CO), bilayer films (SA/[CS–CO]), and multilayer films (SA/[CS–CO]/SA). The results showed that the multilayer films were the most effective at retaining the antimicrobial active ingredients after 10 days storage, with less than 40% of the cinnamon oil being lost. In contrast, as much as 70% of the cinnamon oil was lost from the monolayer films.

The presence of the control layer in multilayer sandwich-like films is important for inhibiting the burst release of active ingredients from packaging. Without the control layer, the active ingredient would be directly in contact with the food surfaces and therefore rapidly diffuse into the food, which would reduce its action time. The control layer, located between the active ingredient and the food surface, is a layer through which the active ingredient should pass to reach the food surface. Therefore, the thickness, chemical composition, and diffusion properties of the control layer are important factors related to the release of active ingredients in these multilayer structures^6^. As an example, silver nanoparticles (AgNPs) have been incorporated into multilayer films assembled from chitosan and graphene (GO) layers: CS/[GO@AgNPs]/CS^69^. This study showed that the release of the nanoparticles from the multilayer films was considerably slower than from monolayer films (GO@AgNPs). Catechins have also been placed within the middle layer of biodegradable multilayer packaging, so that they are not directly in contact with the surfaces of the food, which can extend their antioxidant activity under aerobic conditions and reduce the rancidity of packaged food^70^.

### Composite packaging

The active agents are dispersed within a polymer matrix in composite packaging. The retention and release of the active molecules can then be controlled by altering the interactions between the active and matrix molecules, as well as by the properties of the polymer network^71^. In general, this kind of system can be divided into two types (network-limited system and interaction-limited system) depending on the nature of the dominant release mechanism. For a network-limited system, the release of the active ingredients mainly depends on the properties of the three-dimensional polymer network inside the packaging material, such as its porosity and stability to environmental changes. For an interaction-limited system, the release of the active ingredients mainly depends on the strength of the attractive and repulsive interactions between the active molecules and the polymer matrix.

The molecules in packaging are cross-linked by covalent or noncovalent bonds to form a three-dimensional polymer network structure^25,72^. These cross-links are usually formed by physical, chemical, or enzymatic mechanisms. Cross-linking can be carried out during or after the film structure is formed^73^. Typically, active molecules form cross-links with the matrix molecules during the preparation of film or coating materials. The formation of these cross-links can be used to control the retention and release of the active ingredients in packaging. A number of physicochemical mechanisms can be utilized to control the release profile including: (1) controlling the pore size of the original polymer network; (2) altering the swelling properties of the polymer network; (3) reducing the biodegradation and erosion of the polymer network; (4) controlling the physical or chemical binding of the active ingredient to the polymer network^6,73–77^. As an example, lactoferrin (an antimicrobial protein) has been incorporated into composite packaging assembled by cross-linking gelatin with oligomeric proanthocyanidins (OPCs)^78^. The observed initial burst release of lysozyme from the packaging could be reduced by increasing the OPCs concentration so as to induce greater cross-linking of the polymer network. In addition, it was reported that the OPCs restricted lysozyme release much less in acidic and alkaline environments than in neutral ones, suggesting that these packaging may be used for the pH-triggered release of lysozyme.

In another study, the effect of different concentrations of cinnamaldehyde on the release of lysozyme from gliadin films was studied, where the cinnamaldehyde was used as a cross-linking agent and glycerol was used as a plasticizer^79^. At pH 2, gliadin films containing 1.5%, 3%, and 5% cinnamaldehyde released 48%, 18%, and 8% of the active ingredient after 115 h, respectively. This result was attributed to greater cross-linking of the polymer network at higher cinnamaldehyde concentrations, which would have reduced the pore size and inhibited lysozyme diffusion. In a different experiment, biodegradable active packaging containing maqui berry fruit extract (an antioxidant) were prepared by cross-linking methylcellulose with glutaraldehyde^80^. The quantity of polyphenols released decreased as the cross-linking agent concentration increased, which can again be attributed to a reduction in the pore size of the polymer network.

For interaction-limited systems, the controlled release of active ingredients mainly depends on the covalent and noncovalent bonds formed between the active and matrix molecules. Generally, covalent bonds are stronger than noncovalent ones and therefore lead to a stronger attachment of the active molecules to the polymer matrix. A method has recently been developed that uses covalent bonds to connect active molecules to polysaccharides and then apply them in biodegradable coatings^81^. In this study, a covalent bond was formed through a Schiff base and reductive amination reaction, which combined vanillin, trans-cinnamaldehyde, and chitosan to form a biodegradable coating with good adhesion. The results showed that the formation of a covalent bond overcomes the dissolution problem of lipophilic vanillin and cinnamaldehyde in the reaction, as well as reducing their volatility. The resulting biodegradable coating exhibited better antibacterial activity and shelf-life extension of melon than the control groups. The authors concluded that the advantages of using covalent bonds rather than noncovalent ones are: (1) solubility problems associated with binding active lipophilic reagents in aqueous media can be overcome; (2) the release of volatile compounds can be inhibited; (3) coating adhesion to the fruit can be improved. Thus, covalent bonds are highly effective at limiting the release of active molecules. Other authors have also demonstrated that covalently attaching active molecules to matrix molecules is effective at restricting the release of active ingredients^82,83^. Nevertheless, there is clearly a need for more research on the utilization of both covalent and noncovalent bonds specifically designed to modulate the release profile of active ingredients from biodegradable composite packaging in a more systematic fashion.

## Properties of advanced packaging materials

Compared to petroleum-based packaging materials, biodegradable films that are composed of biopolymers usually have a worse barrier and mechanical properties. Consequently, researchers are looking at approaches to address this problem, including adding crosslinkers, plasticizers, and fillers to the films, as well as changing their structural organization.

### Barrier properties

The barrier properties are an important part of food packaging materials, such as their water vapor permeability, gas permeability, and light transmittance properties. The main parameters affecting mass transfer of substances across packaging materials are diffusivity, solubility, and permeability, which are closely related to the composition and structure of the polymer matrix^84^.

Research has shown that multilayer structures can improve the barrier properties of biodegradable packaging materials. For instance, the mechanical strength and gas barrier properties of multilayer films (PLA/FG/PLA) and monolayer films (FG) assembled from polylactic acid (PLA) and fish gelatin (FG) were compared^85^. The oxygen permeability and water vapor permeability of the three-layer films were 8 and 11 times lower than those of the monolayer films, respectively. Moreover, the tensile strength of the three-layer films increased by around 17.5 MPa, which was appreciably higher than that of the monolayer ones. In another study, starch/PCL-carvacrol/starch multilayer films were prepared by electrospinning using starch and polycaprolactone (PCL) as the matrix materials and carvacrol as the active ingredient^86^. The water vapor permeability of these multilayer films (190 g m^−1^ pa^−1^ s^−1^) was considerably lower than that of starch films (570 g m^−1^ pa^−1^ s^−1^), which highlights the efficacy of the multilayer structures to improve the barrier properties of packaging materials.

Research has also been carried out on composite active packaging materials. For example, biodegradable films have been prepared by incorporating eugenol (active ingredient) into starch and chitosan mixtures (matrix materials) using solvent-casting and solvent-evaporation methods^87^. Optimization of the matrix composition was shown to decrease the WVP of the films from around 2.00 × 10^−10^ g m^−1^ pa^−1^ s^−1^ to 1.29 × 10^−10 ^g m^−1^ pa^−1^ s^−1^. Moreover, the addition of eugenol significantly improved the flexibility, hydrophobicity, antimicrobial activity, and antioxidant properties of the films. The authors obtained information about the molecular interactions between the active ingredient and matrix materials including the hydrogen bonding or other covalent bonding.

### Mechanical properties

The mechanical properties of packaging materials are critical for many of their applications, including the elongation at break, tensile strength, yield stress, yield strain, and Young’s modulus^12^. The layer structure of packaging materials has been shown to have an important effect on their mechanical properties^88^. The overall mechanical properties of multilayer systems depend on the properties of the individual layers they are comprised of, which are usually proteins and/or polysaccharides in biodegradable packaging materials^49,89^. For instance, kafirin/kafirin-gelatin/gelatin films have been prepared using a layer-by-layer solvent-casting method^90^. These multilayer films were shown to have a higher tensile strength (6.3 MPa) and better resistance to water migration than blended films made from kafirin and gelatin.

The mechanical properties of biodegradable films are also related to the interactions between the molecules, which may be enhanced by the incorporation of some active ingredients. As an example, the impact of citric acid concentration on the properties of potato starch-chitosan (PS–CS) composite films prepared by a solution blending-casting method have been examined^91^. Citric acid was found to act as a cross-linker, which significantly enhanced the mechanical properties of the composite films. The performance of the composite films was found to depend on the citric acid concentration used. When the amount of citric acid added was increased from 0 to 15%, the tensile strength of the films increased from around 9.7–12.6 MPa, but when it was increased further the tensile strength decreased. This effect may be due to the plasticizing effect of the citric acid at high concentrations, which reduced the attractive interactions between the matrix molecules. In some studies, it has been reported that incorporation of active ingredients can decrease the mechanical strength of biodegradable films. For example, incorporation of apple polyphenols into chitosan films reduced the tensile strength and elongation at break, but increased the antibacterial and antioxidant properties^92^. Consequently, it is important to elucidate the impact of specific additive ingredients on the mechanical properties of specific biopolymer matrices when developing effective active biodegradable packaging materials.

### Degradability

In many published studies, the characterization of active packaging materials mainly focuses on their bioactivity but neglects their degradability^45^. In general, the degradation of packaging materials may occur through various mechanisms, which can be classified as photodegradable, oxidatively degradable, biodegradable, and hydrolytically degradable^93^. It should be noted, however, that different types of packaging materials are affected differently by their environment, such as pH, temperature, and humidity. Consequently, the rate and extent of the degradation of packaging materials may depend on precisely where they are disposed, *e.g*., a hot humid country near the Equator or a cold dry country near the Artic circle. Indeed, the degradation of many of the proteins and polysaccharides used to assemble packaging materials is strongly affected by pH, temperature, and humidity. Consequently, it is important to establish the degradation of a new packaging material under the conditions it will be disposed of.

## Application of advanced packaging materials in preservation of fruits and meats

Fresh fruits, vegetables, and meat products are easily spoiled due to their high moisture content, so food packaging is needed to extend their shelf life, improve their quality, and ensure their safety. Compared to plastic packaging materials, advanced biodegradable packaging can include active ingredients that have antibacterial and antioxidant functions, thus providing a substantial advantage in extending the quality, shelf life and safety of packaged food.

### Multilayer packaging

In general, multilayer packaging materials can be designed to have better performance, such as enhanced barrier properties, mechanical strength, and protective properties. In particular, active ingredients (such as antioxidants and antimicrobials) can be added to one or more layers of packaging materials to enhance their ability to protect food^94^.

Studies have shown that adding antibacterial agents to multilayer active materials can increase the storage time and the quality of food. For instance, biodegradable composite packaging has been developed using a layer-by-layer assembly technique to prepare multilayer films containing chitosan and polyvinyl alcohol^95^. The performance of these films could be enhanced by adding Cu_2_O-based antibacterial particles to the matrix. These films improve the quality attributes of cherry tomatoes during storage by reducing texture and moisture loss^96^. In another study, multilayer biodegradable films were created to control the respiration of fruits and vegetables by releasing 1-methylcyclopropene (1-MCP) to inhibit ethylene production. The films consist of hydrophobic ethylcellulose as the outer layer, 1-MCP and palladium carbon (1-MCP-Pd/C) as the middle layer, and a hydrophilic hydrogel as the inner layer. The inner hydrophilic gel was designed to absorb excess water from the fruits and vegetables, thereby avoiding the accumulation of condensed water. The inner hydrogel permeability was changed after absorbing moisture, which promotes the diffusion of antiseptic and antibacterial inner components. The film was then applied to mushroom preservation, which delays softening, browning, and weight loss during storage^97^.

Meat products are rich in proteins and lipids, which makes them susceptible to chemical and microbial degradation. Food packaging materials have been developed to overcome these problems by including antibacterial and antioxidant ingredients. For instance, tea polyphenol has been incorporated into multilayer films formed from poly(L-lactic acid), polyvinyl alcohol, and poly (ε-caprolactone) using a lamination technique, which can act as an antioxidant and antimicrobial that increased the shelf life and quality of packaged meat products^98^.

### Composite packaging

There have also been many examples of the application of composite packaging materials for food applications. In this section, we discuss a few examples to highlight the potential of these kinds of materials. A composite film fabricated from egg proteins and cellulose nanomaterials has been shown to increase the shelf lives of banana, avocado, papaya, and strawberry^99^. Films prepared from polyvinyl alcohol, ethylcellulose and tea polyphenols (PVA/EC-TP) using an electrostatic spinning technology were shown to extend the shelf life of packaged pork by 3 days, which was mainly attributed to the antioxidant and antimicrobial activity of the polyphenols^100^. The shelf life of packaging food has also been increased by controlling gas penetration using composite materials assembled from organic porous chitosan microspheres embedded in a poly(L-lactic acid) matrix^95^. The microspheres were used as gas “switches” to regulate the permeability of the films to O_2_, CO_2_, and H_2_O and the selectivity of CO_2_/O_2_. This packaging material was then applied to citrus, mango, cherry, waxberry, and strawberry to form coatings or films. The results show that the films/coatings could prolong the shelf life and improve the quality of the fruits.

## Future trends

Due to environmental pollution and non-renewable petroleum resources, it is likely that researchers will continue to investigate and develop advanced biodegradable materials to replace plastic ones. At present, the share of biodegradable packaging materials in the food packaging market is very low. Indeed, many of these advanced materials are still in the research and development stage, and feasibility studies of their commercial viability have not been performed. To further advance the use of active biodegradable materials, the following problems need to be addressed:

1. The materials and equipment required to manufacture active biodegradable packaging materials should be available, affordable, and capable of large-scale economic production. Many of the materials and processes described in the scientific literature do not currently meet these requirements;
2. The performance of new packaging materials in real situations needs to be systematically addressed. Many of the materials described in the literature may be difficult to apply to real food products, may adversely affect their quality attributes, or may not survive during storage, distribution, and utilization;
3. Government regulations need to be developed globally to address the appropriate creation and utilization of novel kinds of packaging materials.
4. Consumers need to be educated about the potential advantages of new packaging materials to the environment.

In particular, it is important to address the amount of advanced packaging required to ensure that it can meet the market demand. The global output of plastics was around 368 million tonnes in 2019 (https://www.plasticseurope.org/en/resources/market-data). Demand for plastic is on the rise especially with the development of takeout and express delivery services. However, the growth in plastic production has been matched by poor recycling, such as in the case of the European Union, which produced about 61.8 million tonnes of plastic in 2018, while only 9.4 million tonnes were recycled (https://www.plasticseurope.org/en/resources/market-data). As a result, it will be important that any new packaging materials can be produced on these scales if it is going to have a significant environmental benefit. As mentioned earlier, many of the methods developed to produce biodegradable packaging materials in the literature are not currently appropriate to meet these large demands. For instance, many researchers use solvent-casting to produce advanced packaging materials, which are unsuitable for large-scale industrial production. In addition, the active ingredients used in many studies (such as polyphenols, essential oils, or nanofibers) are expensive or have practical challenges to their utilization. For instance, essential oils may evaporate or chemically degrade during storage, thereby reducing their activity. Consequently, a better understanding of the performance of advanced packaging materials under real conditions is required. Thus, there is a need to develop more commercially viable methods for producing biodegradable active packaging materials on a large scale, as well as to rigorously test their performance under realistic application conditions.

Countries around the world are introducing policies to regulate the use of petroleum-based plastics, so as to reduce their negative environmental impacts, such as the European Committee for Standardization (EN13432-2000), American Society for Testing and Materials (ASTM D6400-2004), and the Deutsches Institut für Normung (DIN V 54900). As an example, some of the common standards that have been developed for biodegradable plastics are: (1) passing an aerobic biodegradation test; (2) passing a composting and biodegradation test; (3) passing an ecological non-toxic test; (4) passing a heavy metal content test. New biodegradable materials should also be specifically designed to pass all of these tests. Although the use of active ingredients in food has gradually been regulated in recent years, there is still no unified authority to issue relevant specifications for active biodegradable packaging, and there is still a long way to go in market supervision.

Consumer acceptance of advanced packaging is also an important factor in their promotion. Surveys have shown that the price and quality of food are more important than green packaging, and consumers are only willing to pay a small premium for biodegradable packaging (https://www.plasticseurope.org/en/resources/market-data). To improve consumer acceptance, it will therefore be important to reduce the costs of new biodegradable packaging materials, create government policies (such as taxes and subsidies) that reduce the price differential between biodegradable and non-biodegradable packaging materials, and to inform consumers of environmental benefits.

## Conclusions

There is growing interest in the design and production of biodegradable packaging to replace synthetic plastics so as to reduce the negative environmental impacts associated with their production and disposal. In this review, we focus on biodegradable packaging materials with either multilayer or composite structures. In particular, we focus on the preparation and release mechanisms of these kinds of materials. In addition, the basic release mechanisms of controlled release packaging and the potential impact of active ingredients on the mechanical and barrier properties of films and coatings are reviewed. At present, active biodegradable packaging materials have been successfully designed, characterized, and applied in research laboratories. However, many factors still need to be addressed before the widespread commercial success of these materials, including demonstrating their efficacy in real-life applications, developing large-scale economic manufacturing processes, establishing appropriate government regulations, and gaining consumer acceptance.

## Data Availability

Data sharing is not applicable. This is a review article and no new datasets were generated or analyzed during this article.
